# Effects of 28 days of beta-alanine and creatine supplementation on muscle carnosine, body composition and exercise performance in recreationally active females

**DOI:** 10.1186/s12970-014-0055-6

**Published:** 2014-11-30

**Authors:** Julie Y Kresta, Jonathan M Oliver, Andrew R Jagim, James Fluckey, Steven Riechman, Katherine Kelly, Cynthia Meininger, Susanne U Mertens-Talcott, Christopher Rasmussen, Richard B Kreider

**Affiliations:** Department of Sports Medicine and Nutrition, School of Health and Rehabilitation Sciences, University of Pittsburgh, Pittsburgh, PA 15260 USA; Kinesiology Department, Texas Christian University, Fort Worth, TX 76129 USA; Department of Exercise & Sport Science, University of Wisconsin – La Crosse, La Crosse, WI 54601 USA; Department of Health and Kinesiology, Muscle Biology Laboratory, Texas A&M University, College Station, TX 77843-4243 USA; Department of Health and Kinesiology, Human Countermeasures Laboratory, Texas A&M University, College Station, TX 77843-4243 USA; Department of Medical Physiology, Texas A&M Health Science Center, College Station, TX 77843-1114 USA; Department of Nutrition and Food Science, Institute for Obesity Research and Program Evaluation, Texas A&M University, College Station, TX 77843-4243 USA; Department of Health and Kinesiology, Exercise and Sport Nutrition Lab, Texas A&M University, College Station, TX 77843-4243 USA

## Abstract

**Background:**

The purpose of this study was to examine the short-term and chronic effects of β-ALA supplementation with and without creatine monohydrate on body composition, aerobic and anaerobic exercise performance, and muscle carnosine and creatine levels in college-aged recreationally active females.

**Methods:**

Thirty-two females were randomized in a double-blind, placebo-controlled manner into one of four supplementation groups: β-ALA only (BA, n = 8), creatine only (CRE, n = 8), β-ALA and creatine combined (BAC, n = 9) and placebo (PLA, n = 7). Participants supplemented for four weeks included a loading phase for the creatine for week 1 of 0.3 g/kg of body weight and a maintenance phase for weeks 2–4 of 0.1 g/kg of body weight, with or without a continuous dose of β-ALA of 0.1 g/kg of body weight with doses rounded to the nearest 800 mg capsule providing an average of 6.1 ± 0.7 g/day of β-ALA. Participants reported for testing at baseline, day 7 and day 28. Testing sessions consisted of obtaining a resting muscle biopsy of the vastus lateralis, body composition measurements, performing a graded exercise test on the cycle ergometer for VO_2peak_ with lactate threshold determination, and multiple Wingate anaerobic capacity tests.

**Results:**

Although mean changes were consistent with prior studies and large effect sizes were noted, no significant differences were observed among groups in changes in muscle carnosine levels (BA 35.3 ± 45; BAC 42.5 ± 99; CRE 0.72 ± 27; PLA 13.9 ± 44%, p = 0.59). Similarly, although changes in muscle phosphagen levels after one week of supplementation were consistent with prior reports and large effect sizes were seen, no statistically significant effects were observed among groups in changes in muscle phosphagen levels and the impact of CRE supplementation appeared to diminish during the maintenance phase. Additionally, significant time × group × Wingate interactions were observed among groups for repeated sprint peak power normalized to bodyweight (p = 0.02) and rate of fatigue (p = 0.04).

**Conclusions:**

Results of the present study did not reveal any consistent additive benefits of BA and CRE supplementation in recreationally active women.

## Background

Prior research has shown that beta-alanine (β-ALA) supplementation (e.g., 3.2 g/day to 6.4 g/day for 2 to 10-weeks) increases in muscle carnosine levels, with greater increases observed when larger total dosages have been consumed over time [1-8]. It has been suggested that an increase in intramuscular carnosine levels may lead to an enhanced muscle buffering capacity therefore improving performance by limiting the accumulation of hydrogen ions (H^+^) [2,4,6,7,9,10]. The amount of carnosine elevation ranges from around 10% after two weeks [8] to around 80% after ten weeks [10]. A recent review on the ergogenic benefits of β-ALA supplementation suggested bouts of exercise lasting 1–3 minutes seemed to provide the most benefit with mixed results seen during shorter bouts of exercise [11]. There is also some evidence suggesting that β-ALA supplementation may positively affect body composition when combined with training [12], time to exhaustion [5,11], anaerobic exercise markers such as ventilatory threshold (VT) [13], as well as blood lactate levels [14,15]. The effects of creatine monohydrate have been extensively researched over recent years with respects to the effects on anaerobic exercise performance [16]. High intensity exercise bouts require a faster rate of ATP resynthesis, which is most quickly attained by breaking down phosphocreatine (PCr) [17,18]. Creatine monohydrate supplementation (e.g., 20 g/day for 5 to 7-days and 3–5 g/day thereafter) has been shown to increase the muscle creatine and PCr stores to assist in ATP resynthesis during high intensity exercise [19-21].

Given the reported ergogenic value of β-ALA and creatine supplementation, recent studies have begun to examine the combined effects of β-ALA and creatine monohydrate supplementation on anaerobic exercise performance and muscle carnosine and creatine levels. Results have shown improvements in exercise performance variables such as peak oxygen uptake (VO_2peak_), lactate threshold (LT), and time to exhaustion with a combined supplementation strategy [15]. Similarly, Hoffman and colleagues [22] reported that 10-weeks of β-ALA and creatine monohydrate supplementation during resistance-training had both independent and potentially synergistic ergogenic benefits in male strength/power athletes. However, more research is needed to determine whether there may be additive benefit of co-supplementation of β-ALA and creatine monohydrate supplementation during training. Additionally, the majority of beta-alanine and creatine supplementation studies have used males as participants. Therefore, while it is generally understood how beta-alanine supplementation can affect muscle carnosine levels as well as exercise performance in men, less is known how females will respond to beta-alanine supplementation during training [9,13,14,22-24]. Studies that have examined the effects of β-ALA supplementation in women have reported mixed results [25,26]. Therefore, the purpose of this study was to examine the short-term (7-days) and chronic effects (28-days) of β-ALA supplementation with and without creatine monohydrate on body composition, aerobic and anaerobic exercise performance, and muscle carnosine and muscle creatine levels in college-aged, recreationally-active females. We theorized that co-supplementation of β-ALA and creatine monohydrate may lead to greater ergogenic and performance adaptations by synergistically enhancing anaerobic and/or aerobic capacity.

## Methods

### Experimental design

**Figure 1:**
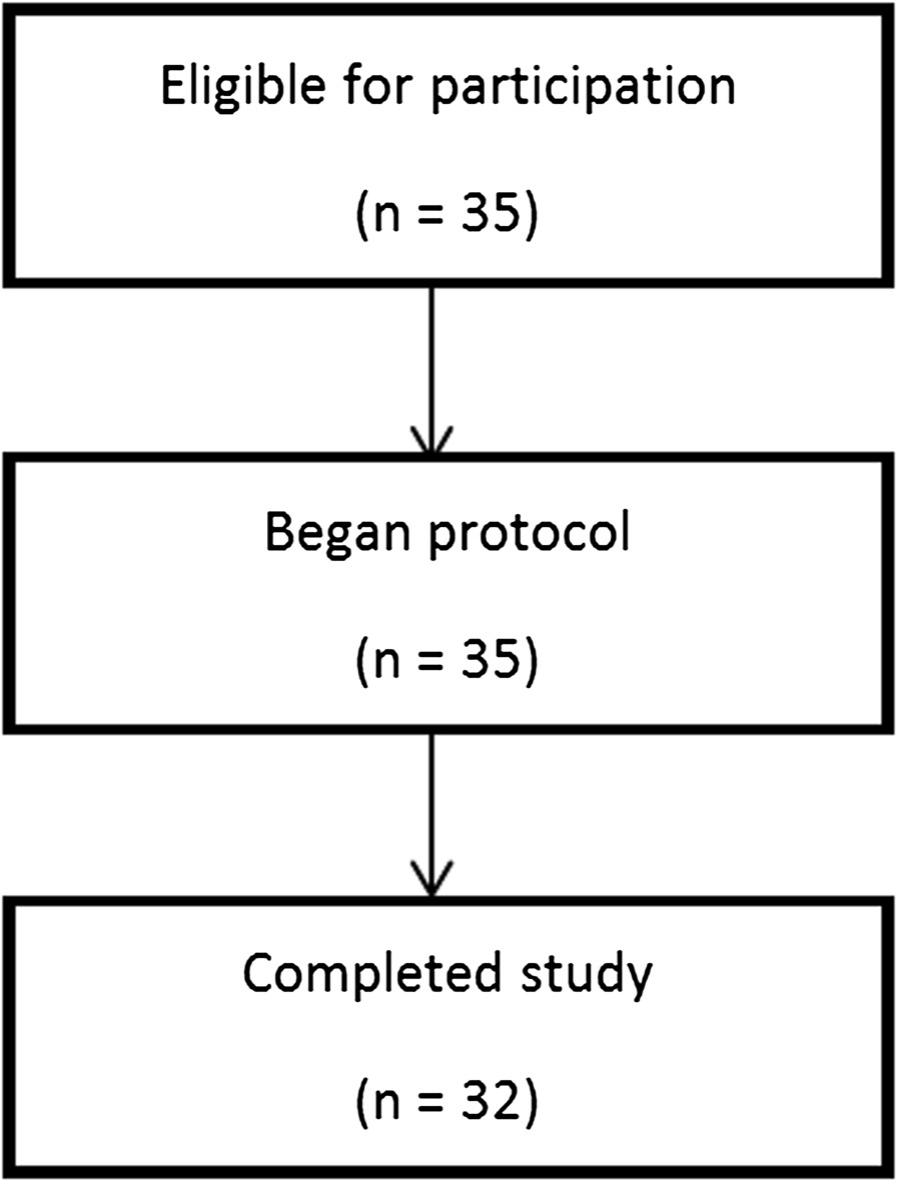
**Consort diagram of study enrollment.**

The present study was a randomized double-blind, placebo-controlled trial that recruited apparently healthy, moderately active females between the ages of 18 and 35 years to participate in the study. Moderately active was defined as having a consistent recent history of participating in exercise (e.g., running, cycling, swimming, resistance training, fitness classes, etc.) for at least 30 minutes per day for 3-days per-week for at least 3 months. Participants were not allowed to participate if they had taken ergogenic levels of nutritional supplements that may have affected muscle mass or anaerobic exercise capacity (i.e. creatine, β-ALA, ergogenic levels of nutritional caffeine, etc.) for at least three months prior to the start of the study. Those who met entrance criteria attended a familiarization session during which time they were familiarized to the study protocol with verbal and written explanations of the study requirements. Participants still interested and meeting the entrance criteria signed informed consent statements in compliance with the Human Participants Guidelines of Texas A&M University and the American College of Sports Medicine. During the familiarization session, participants were also weighed using a standing scale and asked to perform a practice Wingate exercise test on the cycle ergometer at 75% maximal effort. Participants were instructed to find a comfortable seat height and handle bar position which was recorded for subsequent testing sessions. Following the practice Wingate test they were given guidelines to follow for maintaining and recording physical activity during their involvement in the study and scheduled for all subsequent testing sessions and randomly assigned to one of four supplementation groups. Figure 1 shows a consort diagram of those found eligible, initiated, and completed the study protocol. Individuals who did not complete the study did so for reasons unrelated to the study protocol.

### Resting and exercise testing

**Figure 2:**
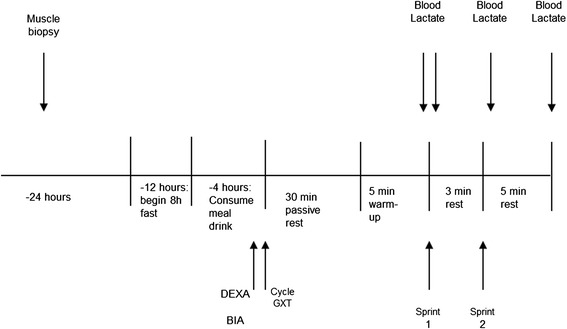
**Outline of testing sessions.**

Resting and exercise testing was performed at baseline prior to any supplementation, at one week of supplementation, and after four weeks at the completion of the study. Figure 2 outlines the events of the testing sessions. Participants were asked to abstain from exercise for 24 hours and fast for at least eight hours prior to baseline testing. One day prior to exercise testing, participants received a percutaneous muscle biopsy from the vastus lateralis muscle of the right leg using standard procedures for the Bergstrom method [27]. Muscle samples were immediately frozen at −80° until analyzed.

The morning after the biopsy, participants were asked to fast for at least eight hours before being asked to consume a standard meal replacement drink (*Boost*® *Original, Nestlé S.A., Vevey, Switzerland*) four hours before reporting to the lab in order to standardize nutritional intake prior to exercise testing. Once reporting to the lab, they were weighed using a free standing scale (*Cardinal Detecto Scale Model 8430, Webb City, Missouri*) and had body composition determined using a Dual Energy X-Ray Absorptiometer (DEXA) [*Discovery QDR Series, Hologic Inc., Waltham, MA*]. Quality control calibration procedures were performed on a spine phantom (*Hologic-X-CLAIBER Model DPA/QDR-1 anthropometric spine phantom*) and a density step calibration phantom prior to each testing session. Mean test-retest reliability studies performed on athletes in the lab had yielded mean coefficients of variation for total bone mineral content and total fat free/soft tissue mass of 0.31% to 0.45% with a mean intra-class correlation of 0.985 [28]. They then had their total body water (TBW) measured using bioelectrical impedance analysis (*ImpediMed DF50, San Diego, CA*). Following the resting measures, participants began exercise testing starting with a maximal graded exercise test (GXT) using an incremental protocol on the Lode Excaliber Sport 925900 cycle ergometer (*Lode BV, Groningen, The Netherlands*) with metabolic measurements recorded on the ParvoMedics True One 2400 Metabolic System (*ParvoMedics, Sandy, Utah*). The protocol began at 50 W maintaining 70 rpm and the intensity was increased by 25 W every three minutes until a pedaling rate of 70 rpm was no longer maintained. Previous research has indicated a mean intra-class correlation of 0.994 and a mean intra-class coefficient of variation of 4.7% when using the ParvoMedics True One 2400 Metabolic system [29]. Calibration procedures were completed prior to each testing session.

Blood samples were taken from the fingertips in the final minute of each stage of exercise and five minutes into the recovery to determine LT. Lactate was determined using a Lactate Scout (*Sports Resource Group, USA*) handheld analysis device. Previous research has yielded a mean intra-class correlation of 0.91 and a mean intra-class coefficient of variation of 10.2% [30]. Calibration procedures were completed prior to each testing session. The LT was calculated two different ways including the point at which blood lactate concentrations rises more than 1.0 mM/l from the previously recorded value LT and the point at which blood lactate level was greater than or equal to 4.0 (also termed the onset of blood lactate, OBLA). All values were reported as a percent of VO_2peak_. The ventilatory threshold (VT) was determined as the point during the GXT where pulmonary ventilation increased at a disproportional rate with VO_2_, and was also recorded as a percent of VO_2peak_. Following the GXT, participants rested passively for 30 minutes and then performed two 30-sec Wingate Anaerobic Capacity Tests at a standardized work rate of 7.5 J/kg/rev. The seat position was standardized between trials and the participant was asked to pedal as fast as possible prior to application of the workload and sprint at all-out maximal capacity during the 30-second test with 3 minutes of passive rest in between. Blood was taken from the fingertips before the start of Wingate 1, immediately following Wingate 2, and after 5 minutes of passive recovery following the completion of both Wingate tests. Test-to-test variability in performing repeated Wingate anaerobic capacity tests in our laboratory yielded a coefficient of variation (CV) of ±15% with a test retest correlation of *r* = 0.98 for mean power [31]. Participants practiced the anaerobic capacity test during the familiarization session to minimize learning effects.

### Supplementation protocol

The supplementation protocol was modified from those used by Hoffman et al. in 2006 [22] and Zoeller et al. in 2007 [15]. The creatine monohydrate (*Creapure*®*, AlzChem Trostberg GmbH, Germany*) supplementation was provided in the form of a powder that the participants were instructed to mix with water (6–10 oz.). The β-ALA used in this study (*CarnoSyn*®*, Natural Alternatives International, Inc., San Marcos, CA*) was a sustained release form of β-ALA that was provide in 800 mg capsules. In a double blinded manner, participants were matched to body mass and randomly assigned to either ingest β-ALA (BA, n = 8), creatine monohydrate (CRE, n = 8), a combination of β-ALA and creatine monohydrate (BAC, n = 9), or a placebo (PLA, n = 7). The β-ALA only group received a dose of 0.1 g/kg body weight per day for the entire 28 days with 0.3 g/kg/day of dextrose for week 1 and 0.1 g/kg/day of dextrose for weeks 2–4. The creatine only group was given a dose of 0.3 g/kg/day of creatine for week 1 and 0.1 g/kg/day for weeks 2–4, with 0.1 g/kg/day maltodextrin for the 28 days. The β-ALA and creatine combined group consumed a 0.1 g/kg/day of β-ALA for the entire 28 days (about 6.1 ± 0.7 g/day) with 0.3 g/kg/day of creatine for week 1 (about 18 ± 1.8 g/day) and 0.1 g/kg/day of creatine for weeks 2–4 (about 6.1 ± 0.7 g/day). Finally, the placebo group was given 0.1 g/kg/day of maltodextrin capsules for all 28 days with 0.3 g/kg/day of dextrose powder for week 1 and 0.1 g/kg/day for weeks 2–4 which served as the placebo for creatine monohydrate. The β-ALA and matched placebo doses were rounded to the nearest 800 mg capsule while the creatine monohydrate and matched placebo doses were rounded to the nearest 0.1 g. The rationale in providing more of a relative dosage of β-ALA was an attempt to help normalize the administration β-ALA to body mass to doses that are commercially available. Participants were instructed to take divided doses of the supplements at 4 intervals throughout the day with water and/or food, as close to 8:00 am, 12:00 pm, 4:00 pm and 8:00 pm as possible. Participants were given supplements one week at a time and were asked to return the empty containers to ensure compliance. They also completed supplementation logs each week to monitor compliance of supplementation.

### Muscle analysis

The muscle samples were obtained using a modified Bergstrom muscle biopsy technique and were analyzed for phosphocreatine (PCr) and creatine (Cr) content based on methods from previous studies [32-34]. Percutaneous muscle biopsies (50–70 mg) were obtained from the middle portion of the vastus lateralis muscle of the thigh at the midpoint between the patella and greater trochanter of the femur. The biopsy needle was inserted 1–2 cm into the muscle prior to tissue extraction. Once the biopsy was taken, adipose tissue was trimmed from the muscle specimens and then the muscle sample was immediately frozen in liquid nitrogen and stored at −80°C for later analysis. A total of three muscle samples were obtained, two from one leg and one from the opposite leg (Day 0, 6, & 27). The left leg was used for pre and post biopsies with the right leg being used for the mid biopsy.

Muscle tissue samples were analyzed spectrophotometrically in duplicate to determine PCr and Cr content using methods developed by Harris and colleagues [20,21,32]. Briefly, approximately 50–70 mg of muscle tissue was cut and placed in a microfuge tube, and then placed in a vacuum centrifuge (*Savant ISS110 SpeedVac Concentrator, Thermo Scientific, Milford, MA*) and centrifuged for 18–24 hours. Connective tissue was removed from the dried samples which were then ground into a powder in a porcelain plate and placed into pre-weighed microfuge tubes. Muscle metabolites were extracted in a 0.5 M perchloric acid/1 mM EDTA solution on ice for 15 minutes, while periodically vortexing. Samples were then centrifuged at 7,000 rpm for 5 minutes. The supernatant was transferred into a pre-weighed microfuge tube and neutralized with 2.1 M KHCO_3_/0.3 M MOPS solution. The samples were then centrifuged again at 7,000 rpm for 5 minutes and the supernatant was removed and placed into microfuge tubes and frozen at −80°C.

Extracts were assayed for PCr in the presence of 50 mM Tris buffer, pH 7.4; 1 mM magnesium chloride, 0.5 mM dithiothreitol, 100 μM glucose, 50 μM NADP^+^, 350 U/mL glucose-6-phosphate dehydrogenase. The assay was carried out in 13 × 75 glass screw-top tubes using 10 μL of sample to 1 mL of reagent. The reactant solution was vortexed and read using a fluorometer (*Shimadzu RFMini 150, Japan*) with an excitation wavelength of 360 nm and an emission wavelength of 460 nm. Twenty five mL of hexokinase solution was added to 1 mL of reagent and stabilized. For PCr, 20 μL of creatine kinase/ sulfodicholorphenol (CK/SDP) solution was added to the tubes, which were vortexed and incubated in a dark at room temperature for 60 minutes when samples were read again for post-reaction absorbance values.

Extracts were assayed for Cr in the presence of 50 mM imidazole buffer, pH 7.4; 5 mM magnesium chloride; 20 mM potassium chloride; 25 μM phosphoenolpyruvate; 200 μM ATP; 45 μM NADH; 1250 U/mL lactate dehydrogenase; 2000 U/mL pyruvate kinase. The assay was carried out in a standard fluorescence microplate reader using 10 μL of sample to 1 mL of reagent. The reactant solution was vortexed and read using a fluorometer (*Shimadzu RFMini 150, Japan*) with an excitation wavelength of 340 nm and an emission wavelength of 460 nm for baseline absorbance values. Five μL of CK (25 μ/mg) was added to 1 mL of the above buffer and stabilized using 1 mL of reagent. After 10 minutes the plate was read again for post-reaction absorbance values. Test to test reliability of duplicate muscle creatine assays was 0.22 ± 2.4% (r = 0.90) with a coefficient of variation of 6.8%. Creatine and PCr were analyzed using a SpectraMax 250 (*Molecular Devices, Sunnyvale, CA*). All results were expressed as mmol/kg dry weight (DW). Total muscle creatine content was calculated by adding the resulting amounts of PCr and Cr content together.

Muscle carnosine was analyzed using the HPLC procedures developed by Dunnett and Harris [35]. The muscle samples were prepared using the same drying methods as before. Muscle samples were analyzed using an Aquity-UPLC system (*Waters, Milford, MA*). Chromatography was performed using a Thermo Scientific Hypersil ODS (150 mm × 4.6 mm ID) analytical column protected by a Hypersil ODS guard column. Solvents were filtered to 0.45 μm. Compounds were eluted using a solvent gradient at ambient temperature with the following mobile phases: LINE A: Solvent A:20 mM phosphate buffer [(20 mM Na_2_HPO_4_ (2.84 g/l) + 20 mM NaH_2_PO_4_.2H_2_O (3.12 g/l)], pH 6.8 – tetrahydrofuran (995:5 v/v); LINE B: Solvent B: 20 mM phosphate buffer, pH 6.8 – methanol - acetonitrile (500:350:150, v/v); LINE C: 100% methanol; LINE D: 100% water; 2 liters 20 mM Na_2_HPO_4_ = 5.68 g; 2 liters 20 mM NaH_2_PO_4_.2H_2_O = 6.24 g.

### Statistical analysis

Data were analyzed using SPSS 20.0 software (*IBM, Chicago, IL*). Missing data, if any, were replaced using the last observed value or series mean [36]. One-way Analysis of Variance (ANOVA) was used to analyze baseline demographic data. Multivariate Analysis of Variance (MANOVA) with repeated measures was used to analyze logically-related variables. The Wilks’ Lambda time and group × time p-levels were used to assess the overall MANOVA effects. Univariate tests from the MANOVA are presented to show individual variable results. In some instances, quadratic interaction p-levels are reported indicating that non-linear but significant differences were observed among groups over time. On select variables, delta values or percent change values were calculated and analyzed by ANOVA with repeated measures in order to evaluate the change in values from baseline. Data were considered statistically significant when the p-value was less than 0.05 while trends were considered when p-values ranged between 0.05 and 0.10. Tukey’s least significant difference (LSD) post hoc analyses were used to determine where the significance was obtained. Cohen’s *d* calculations for effect size were performed to assess magnitude of effect. Non-significant data that showed moderate to large effects sizes were also noted as trends for follow-up with larger sample populations. Data are presented as means ± standard deviation, except group means were presented ± standard error of the mean.

## Results

A total of 32 apparently healthy, recreationally active females completed the protocol for the present study. Participants were 21.5 ± 2.8 years, 60.5 ± 6.1 kg, 40.2 ± 3.8 kg fat free mass, and 26.7 ± 5.8% body fat. One-way ANOVA analysis revealed no significant differences (p > 0.05) between groups at baseline for age, weight, fat free mass, body fat percentage, muscle carnosine, or muscle phosphagen levels.

### Muscle carnosine and phosphagen levels

Table 1 presents muscle carnosine and phosphagen levels observed in the present study. Muscle samples were obtained from 31 total participants. There was sufficient sample to analyze 27 samples for carnosine and 19 samples for phosphagen levels. Repeated measures ANOVA analysis revealed no significant time (p = 0.22) or time × group (p = 0.82) effects among groups in muscle carnosine levels while a significant group effect (p = 0.04) was observed with supplementation. Post hoc analysis revealed that mean muscle carnosine levels in the PLA group were significantly lower than all other groups. One-way ANOVA of percent changes in muscle carnosine levels suggested that those in the BA and BAC groups observed the greatest increase in muscle carnosine levels; however, these apparent differences were not significantly different among groups (BA 35.3 ± 45; BAC 42.5 ± 99; CRE 0.72 ± 27; PLA 13.9 ± 44%, p = 0.59). Repeated measures MANOVA analysis on muscle creatine (Cr), phosphocreatine (PCr), and total creatine (Cr_tot_) content revealed no overall Wilks’ Lamda time (p = 0.22) or time by group (0.80) effects among groups. Univariate repeated measures ANOVA revealed significant quadratic differences among groups in changes in PCr (p_q_ = 0.05). However, differences observed could not be attributed to creatine supplementation. Delta analysis revealed that Cr_tot_ levels increased to a greater degree after one week of loading (BA −3.57 ± 31; BAC 12.04 ± 36; CRE 6.86 ± 26; PLA −3.57 ± 31 mmol/kg DW) but these changes were not maintained during the maintenance period (BA 11.2 ± 17; BAC 0.13 ± 36; CRE −8.23 ± 29; PLA 11.2 ± 17 mmol/kg DW) and no time × group effects were observed among groups (p = 0.79).

**Table 1:** **Muscle carnosine, creatine, phosphocreatine, and total creatine over 4 weeks**

	**BA**	**BAC**	**CRE**	**PLA**	**Time**	**P value**
**Carnosine** (μmol/g)						
Baseline	19.74 ± 8.69	20.81 ± 7.66	20.80 ± 2.81	15.70 ± 4.70	19.27 ± 6.50	T = 0.22
4 Weeks	23.68 ± 1.56	24.23 ± 4.09	21.04 ± 7.00	16.53 ± 4.80	21.37 ± 5.31	G = 0.04
Group	21.71 ± 1.44^d^	22.52 ± 1.34^d^	20.92 ± 1.44^d^	16.12 ± 1.70^abc^		T × G = 0.82
**Creatine** (mmol/kg DW)						
Baseline	47.13 ± 19.88	59.82 ± 37.7	72.96 ± 29.59	59.85 ± 7.79	59.92 ± 27.6	T_q_ = 0.07
1 Week	50.73 ± 26.83	65.49 ± 15.25	88.55 ± 38.72	68.17 ± 7.74	67.80 ± 24.9	G = 0.14
4 Weeks	42.33 ± 16.24	59.90 ± 9.77	67.72 ± 15.94	57.19 ± 8.07	57.28 ± 14.3	T × G = 0.99
Group	46.73 ± 8.26	61.74 ± 6.24	76.4 ± 8.26	61.74 ± 8.26		
**Phosphocreatine** (mmol/kg DW)						
Baseline	22.18 ± 4.28	22.94 ± 18.02	31.69 ± 16.54	21.35 ± 4.44	24.29 ± 13.3	T = 0.10
1 Week	25.91 ± 9.88	32.61 ± 19.62	23.43 ± 4.40	24.08 ± 4.23	27.47 ± 13.0	G = 0.98
4 Weeks	34.75 ± 7.38†^b^	26.87 ± 7.04^a^	30.51 ± 6.26	31.43 ± 9.39	30.25 ± 7.49	T × G_q_ = 0.05
Group	27.61 ± 4.69	27.47 ± 3.54	28.55 ± 4.69	25.62 ± 4.69		
**Total creatine** (mmol/kg DW)						
Baseline	63.04 ± 23.30	82.75 ± 37.19	105.11 ± 26.57	80.76 ± 11.18	82.89 ± 29.8	T = 0.76
1 Week	59.47 ± 27.07	94.79 ± 8.30	111.98 ± 37.99	85.53 ± 4.39	89.02 ± 26.8	G = 0.02
4 Weeks	74.28 ± 11.40	82.89 ± 5.32	96.89 ± 14.61	85.91 ± 18.07	84.66 ± 13.4	T × G = 0.79
Group	65.60 ± 7.65^bc^	86.81 ± 5.78^a^	104.66 ± 7.65^a^	84.07 ± 7.65		

Repeated measures ANOVA was performed on n = 27 (muscle carnosine) and a repeated measures MANOVA was performed on n = 19 (muscle phosphagen) samples. Individual group and time data are presented as means ± SD while time and group effects are presented as means ± SEM. MANOVA analysis on muscle creatine and phosphocreatine levels revealed an overall Wilks’ Lambda time (p = 0.22) and group × time (p = 0.80) effects. Univariate ANOVA p-levels from MANOVA analysis are presented for each variable. BA signifies beta-alanine only group; BAC represents beta-alanine and creatine combined group; CRE represents the creatine only group; PLA represents the placebo group; T represents time p-level; G represents group p-level, and T × G represents interaction. _q_represents quadratic p-level. † represents p < 0.05 difference from baseline. ^a^represents p < 0.05 difference from BA group. ^b^represents p < 0.05 difference from BAC group. ^c^represents p < 0.05 difference from CRE group. ^d^represents p < 0.05 difference from PLA group.

### Body composition

Table 2 presents changes in body composition and body water. A MANOVA was run on body weight and DEXA determined fat mass, fat free mass, and percent body fat. An overall Wilks’ Lamda time effect was observed (p < 0.001) with no significant Wilks’ Lamda time by group effects (p = 0.57). Repeated measures univariate ANOVA analysis revealed significant time effects in changes in body weight (p < 0.01), fat mass (p = 0.05), fat free mass (p < 0.001), and body fat (p = 0.02). However, no significant time × group effects were observed. No significant time (p = 0.79) or time × group (p = 0.36) effects were observed in percent total body water.

**Table 2:** **Changes in body weight, body composition, and body water**

	**BA (n = 8)**	**BAC (n = 9)**	**CRE (n = 8)**	**PLA (n =7)**	**Time**	**P value**
**Body weight** (kg)						
Baseline	63.16 ± 7.48	59.18 ± 5.42	61.15 ± 5.68	58.60 ± 5.81	60.54 ± 6.10	T = 0.01
1 Week	63.32 ± 7.30	59.33 ± 5.32	61.38 ± 5.94	59.22 ± 6.07	60.82 ± 6.11	G = 0.50
4 Weeks	63.60 ± 7.28	60.16 ± 5.02	61.26 ± 5.65	59.44 ± 5.88	61.14 ± 5.91†	T × G = 0.49
Group	63.36 ± 2.14	59.56 ± 2.02	61.26 ± 2.14	59.09 ± 2.29		
**Fat mass** (kg)						
Baseline	16.49 ± 4.93	14.10 ± 3.27	14.30 ± 4.88	14.76 ± 3.94	14.89 ± 4.19	T = 0.05
1 Week	15.52 ± 4.30	13.17 ± 2.57	13.86 ± 4.79	13.84 ± 4.07	14.08 ± 3.88†	G = 0.50
4 Weeks	16.59 ± 4.67	12.99 ± 3.01	13.44 ± 3.00	14.02 ± 4.39	14.23 ± 3.88	T × G = 0.57
Group	16.20 ± 1.38	13.42 ± 1.30	13.89 ± 1.38	14.21 ± 1.47		
**Fat free mass** (kg)						
Baseline	41.08 ± 4.20	39.77 ± 4.42	41.36 ± 3.33	38.40 ± 3.18	40.19 ± 3.35	T = 0.000
1 Week	42.05 ± 3.78	40.86 ± 4.65	42.06 ± 3.17	39.86 ± 3.38	41.23 ± 3.76†	G = 0.59
4 Weeks	41.40 ± 4.35	41.72 ± 4.44	42.55 ± 3.85	39.92 ± 3.09	41.45 ± 3.93†	T × G = 0.17
Group	41.51 ± 1.35	40.78 ± 1.27	41.99 ± 1.35	39.40 ± 1.44		
**Percent fat** (%)						
Baseline	28.23 ± 6.72	26.03 ± 5.25	25.25 ± 6.62	27.51 ± 5.34	26.71 ± 5.84	T = 0.02
1 Week	26.51 ± 5.08	24.40 ± 4.56	24.34 ± 6.52	25.46 ± 5.23	25.14 ± 5.19†	G = 0.62
4 Weeks	28.25 ± 6.34	23.76 ± 5.14	23.88 ± 4.11	25.60 ± 5.94	25.32 ± 5.48†	T × G = 0.45
Group	27.66 ± 1.88	24.73 ± 1.78	24.49 ± 1.88	26.19 ± 2.02		
**Total body water** (%)						
Baseline	51.31 ± 4.09	53.79 ± 6.56	48.86 ± 5.48	50.90 ± 4.48	51.30 ± 5.33	T = 0.66
1 Week	51.27 ± 3.83	52.56 ± 4.22	50.53 ± 3.61	50.01 ± 3.39	51.15 ± 3.72	G = 0.44
4 Weeks	50.61 ± 3.06	53.33 ± 3.96	51.66 ± 3.66	50.53 ± 3.08	51.59 ± 3.66	T × G = 0.30
Group	51.07 ± 1.45	53.23 ± 1.35	50.35 ± 1.47	50.48 ± 1.45		

Individual group and time data are presented as means ± SD while time and group effects are presented as means ± SEM. MANOVA analysis on DEXA body composition revealed overall Wilks’ Lambda time (p < 0.001) and group × time (p = 0.57) effects. Univariate ANOVA p-levels from MANOVA analysis are presented for each body composition variable. BA signifies beta-alanine only group; BAC represents beta-alanine and creatine combined group; CRE represents the creatine only group; PLA represents the placebo group; T represents time p-level; G represents group p-level, and T × G represents interaction. † represents p < 0.05 difference from baseline.

### Aerobic exercise performance

Table 3 presents changes observed among groups in VO_2peak_, time to exhaustion, metabolic equivalents (METS), and ventilatory anaerobic threshold (VANT). MANOVA analysis revealed an overall Wilks’ Lamda time effect (p = 0.049) and time × group effects (p = 0.017). Univariate ANOVA analysis revealed significant time effects in VANT (p < 0.001) with no significant time effects observed VO_2peak_ (p = 0.54), time to exhaustion (p = 0.30), and METS (p = 0.35). Interaction trends were observed among groups in VO_2peak_ (p_q_ = 0.07) and METS (p_q_ = 0.07) with no significant time × group differences observed among groups in time to exhaustion (p_q_ = 0.13) of VANT (p = 0.19). However, post-hoc analysis did not reveal any meaningful changes over time among or between groups.

**Table 3:** **Aerobic exercise capacity results observed among groups**

	**BA (n = 8)**	**BAC (n = 8)**	**CRE (n = 8)**	**PLA (n =6)**	**Time**	**P value**
**VO**_**2peak**_ (ml/kg/min)						
Baseline	41.50 ± 5.60	40.85 ± 6.98	34.20 ± 5.73	35.88 ± 9.65	38.26 ± 7.33	T = 0.54
1 Week	41.58 ± 5.96	39.89 ± 7.62	36.10 ± 6.04	33.75 ± 10.5	38.10 ± 7.72	G = 0.20
4 Weeks	41.53 ± 6.12	39.35 ± 6.96	35.34 ± 2.98	37.90 ± 9.03	38.57 ± 6.52	T × G_q_ = 0.07
Group	41.53 ± 2.35	40.03 ± 2.35	35.21 ± 2.35	35.84 ± 2.72		
**Time to exhaustion** (sec)						
Baseline	1,249 ± 210	1,185 ± 303	963 ± 289	1,093 ± 324	1,125 ± 290	T = 0.30
1 Week	1,294 ± 246	1,196 ± 360	1,020 ± 251	1,032 ± 314	1,142 ± 303	G = 0.24
4 Weeks	1,293 ± 240	1,173 ± 319	1,046 ± 198	1,083 ± 310	1,153 ± 273	T × G_q_ = 0.13
Group	1,279 ± 97	1,185 ± 97	1,010 ± 97	1,069 ± 112		
**METS**						
Baseline	11.88 ± 1.60	11.63 ± 2.00	9.79 ± 1.64	10.27 ± 2.77	10.93 ± 2.09	T = 0.56
1 Week	11.88 ± 1.70	11.41 ± 2.19	10.31 ± 1.74	9.63 ± 3.00	10.89 ± 2.21	G = 0.20
4 Weeks	11.85 ± 1.74	11.25 ± 1.98	10.09 ± 0.85	10.83 ± 2.54	11.02 ± 1.86	T × G_q_ = 0.07
Group	11.87 ± 0.67	11.43 ± 0.67	10.06 ± 0.37	10.24 ± 0.78		
**Ventilatory threshold** (%VO_2peak_)						
Baseline	86.81 ± 8.73	86.44 ± 10.73	77.01 ± 6.46	85.75 ± 10.64	83.89 ± 9.68	T < 0.001
1 Week	84.06 ± 7.34	85.35 ± 10.47	78.61 ± 10.53	84.78 ± 11.03	83.30 ± 9.79	G = 0.44
4 Weeks	78.59 ± 9.75	78.26 ± 13.02	76.50 ± 11.21	75.30 ± 9.63	77.29 ± 10.58†ǂ	T × G = 0.19
Group	83.15 ± 2.92	83.35 ± 2.92	77.38 ± 2.92	82.29 ± 3.73		

Individual group and time data are presented as means ± SD while time and group effects are presented as means ± SEM. MANOVA analysis revealed overall Wilks’ Lambda time (p < 0.049) and group × time (p = 0.017) effects. Univariate ANOVA p-levels from MANOVA analysis are presented for each variable. BA signifies beta-alanine only group; BAC represents beta-alanine and creatine combined group; CRE represents the creatine only group; PLA represents the placebo group; T represents time p-level; G represents group p-level, and T × G represents interaction. _q_ represents quadratic p-level. † represents p < 0.05 difference from baseline. ǂ represents p < 0.05 difference from 1 week.

### Blood lactate and lactate threshold

Blood lactate levels observed during aerobic capacity testing is presented in Table 4. No significant differences were observed among groups in pre-exercise lactate levels. MANOVA analysis revealed no overall Wilks’ Lamda time (p = 0.33) or group × time (p = 0.34) effects. ANOVA univariate analysis revealed a significant time × group interaction (p = 0.05) was observed in peak lactate among groups. Post-hoc analysis revealed that participants in the BA group had a significantly higher baseline peak lactate response than other groups and that peak lactate levels decreased after 4-weeks of supplementation after BA supplementation despite performing similar amounts of work. Participants in the BA group also experienced significantly less change in resting to maximal lactate levels despite performing similar amounts of work after 1 and 4 weeks of BA supplementation. There were no significant differences for lactate threshold between groups or over time.

**Table 4:** **Blood lactate results observed during the maximal exercise test**

	**BA (n = 8)**	**BAC (n = 9)**	**CRE (n = 8)**	**PLA (n =7)**	**Time**	**P value**
**Resting lactate** (mmol/L)						
Baseline	1.44 ± 0.64	1.51 ± 0.44	1.33 ± 0.24	2.03 ± 0.96	1.56 ± 0.63	T = 0.71
1 Week	1.60 ± 0.61	1.43 ± 0.58	1.18 ± 0.28	1.54 ± 0.46	1.43 ± 0.51	G = 0.15
4 Weeks	1.58 ± 0.43	1.36 ± 0.49	1.53 ± 0.47	1.66 ± 0.45	1.52 ± 0.45	T × G = 0.38
Group	1.54 ± 0.12	1.43 ± 0.11	1.34 ± 0.12	1.74 ± 0.13		
**Peak lactate** (mmol/L)						
Baseline	12.91 ± 4.48^bcd^	8.63 ± 3.34^a^	9.71 ± 1.83^a^	7.54 ± 2.26	9.73 ± 3.64	T = 0.10
1 Week	10.43 ± 2.27	8.93 ± 3.18	9.20 ± 2.15	8.76 ± 1.25	9.33 ± 2.36	G = 0.07
4 Weeks	9.85 ± 1.89†	8.04 ± 2.59	8.99 ± 1.46	8.64 ± 2.28	8.87 ± 2.12	T × G = 0.05
Group	11.06 ± 0.77^bd^	8.54 ± 0.73^a^	9.30 ± 0.77	8.31 ± 0.83^a^		
**Lactate threshold** (% Peak VO_2_)						
Baseline	65.18 ± 8.37	67.12 ± 14.08	66.09 ± 7.54	66.40 ± 10.14	66.22 ± 7.45	T = 0.14
1 Week	68.86 ± 5.98	69.92 ± 7.44	67.31 ± 6.64	64.51 ± 6.88	67.82 ± 6/75	G = 0.91
4 Weeks	67.04 ± 7.66	69.04 ± 6.91	69.61 ± 9.42	73.53 ± 5.83	69.67 ± 7.58	T × G = 0.86
Group	67.03 ± 1.73	68.70 ± 1.63	67.67 ± 1.73	68.15 ± 1.85		
**Onset of blood lactate** (%VO_2peak_)						
Baseline	77.50 ± 9.89	82.94 ± 10.9	81.71 ± 8.82	83.81 ± 8.82	81.47 ± 9.54	T = 0.13
1 Week	78.50 ± 10.1	84.90 ± 9.30	83.48 ± 7.67	75.17 ± 17.43	80.82 ± 11.5	G = 0.58
4 Weeks	84.14 ± 8.35	86.68 ± 8.51	83.76 ± 11.68	84.77 ± 11.93	84.90 ± 9.70	T × G = 0.81
Group	80.05 ± 2.61	84.84 ± 2.46	82.98 ± 2.61	81.25 ± 2.79		
**Blood lactate difference from baseline to max** (mmol/L)						
Baseline	11.48 ± 4.19^bcd^	7.10 ± 3.52^a^	8.39 ± 1.75^ad^	5.51 ± 2.15^ac^	8.17 ± 3.67	T = 0.08
1 Week	8.83 ± 2.64†	7.43 ± 3.31	8.03 ± 2.03	7.20 ± 1.59	7.88 ± 2.49	G = 0.06
4 Weeks	8.28 ± 1.88†	6.61 ± 2.73	7.46 ± 1.26	6.81 ± 2.24	7.28 ± 2.12	T × G = 0.02
Group	9.53 ± 0.79	7.05 ± 0.74	7.96 ± 0.79	6.51 ± 0.84		

Individual group and time data are presented as means ± SD while time and group effects are presented as means ± SEM. MANOVA analysis revealed overall Wilks’ Lambda time (p = 0.33) and group × time (p = 0.34) effects. Univariate ANOVA p-levels from MANOVA analysis are presented for each variable. BA signifies beta-alanine only group; BAC represents beta-alanine and creatine combined group; CRE represents the creatine only group; PLA represents the placebo group; T represents time p-level; G represents group p-level, and T × G represents interaction. † represents p < 0.05 difference from baseline. ^a^represents p < 0.05 difference from BA group. ^b^represents p < 0.05 difference from BAC group. ^c^represents p < 0.05 difference from CRE group. ^d^represents p < 0.05 difference from PLA group.

### Anaerobic exercise performance

**Figure 3:**
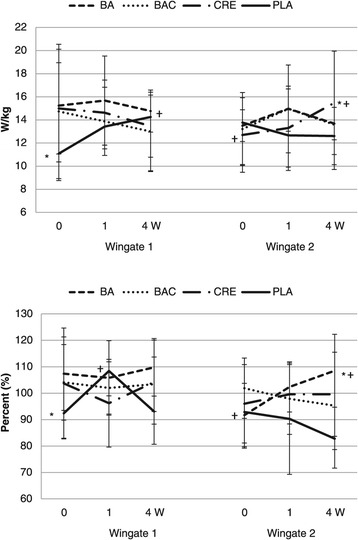
**Wingate anaerobic capacity peak power and rate of fatigue results.** Data are means ± SD. BA signifies beta-alanine only group; BAC represents beta-alanine and creatine combined group; CRE represents the creatine only group; and, PLA represents the placebo group. * represents p < 0.05 difference between PLA and BA, BAC, or CRE groups. † represents p < 0.05 difference from Wingate 1 baseline while ‡ represents p < 0.05 difference from Wingate 2 baseline.

Results from the Wingate anaerobic capacity testing are presented in Table 5 and Figure 3. Peak Power (PP) normalized to body weight demonstrated a significant time × group × Wingate interaction (p = 0.02). Relative mean power, total work, and rate of fatigue significantly decreased from the first to second Wingate anaerobic capacity tests. Significant time × Wingate × group effects were observed among groups in relative peak power (p = 0.02) and rate of fatigue (p = 0.04). Post-hoc analysis demonstrated that placebo relative peak power and rate of fatigue values were lower than other groups at baseline no differences among groups after 1 or 4 weeks of supplementation. Additionally, relative peak power in the second sprint test in the CRE group was significantly greater than PLA values after 4 weeks of supplementation. Rate of fatigue after 4-weeks of supplementation was significant higher in the BA group compared to initial fatigue values when performing the second Wingate test before supplementation.

**Table 5:** **Anaerobic capacity repeated sprint performance results**

	**BA (n = 8)**	**BAC (n = 9)**	**CRE (n = 8)**	**PLA (n =7)**	**Time**	**P value**
**Peak power** (W/kg)						
**Wingate 1**						
Baseline	15.24 ± 4.87^d^	14.74 ± 5.81^d^	15.00 ± 3.95^d^	11.09 ± 2.33^abc^	14.13 ± 4.62	T = 0.67
1 Week	15.68 ± 3.85	13.88 ± 2.95	14.63 ± 2.82	13.43 ± 1.93	14.42 ± 2.97	G = 0.46
4 Weeks	14.77 ± 1.82	13.00 ± 3.40	13.46 ± 2.69	14.26 ± 4.73†	13.83 ± 3.19	T × G = 0.58
**Wingate 2**	G × W = 0.91					
Baseline	13.51 ± 1.37	13.22 ± 3.15	12.70 ± 3.22†	13.77 ± 3.62	13.28 ± 2.83	W = 0.59
1 Week	14.97 ± 1.95	14.96 ± 3.80	13.30 ± 3.38	12.67 ± 3.04	14.05 ± 3.16	T × W = 0.46
4 Weeks	13.68 ± 1.40	13.60 ± 3.89	15.48 ± 4.47	12.61 ± 2.47	13.87 ± 3.33	T × W × G = 0.02
**Mean power** (W/kg)						
**Wingate 1**						
Baseline	6.13 ± 0.97	5.77 ± 0.59	5.87 ± 0.53	5.44 ± 1.21	5.81 ± 0.84	T = 0.46
1 Week	6.25 ± 0.83	5.89 ± 0.65	6.01 ± 0.65	5.39 ± 0.67	5.90 ± 0.74	G = 0.36
4 Weeks	6.07 ± 0.80	5.75 ± 0.61	5.65 ± 0.86	5.69 ± 0.76	5.79 ± 0.74	T × G = 0.48
**Wingate 2**	G × W = 0.89					
Baseline	5.60 ± 0.87	5.42 ± 0.55	5.20 ± 0.54	5.10 ± 1.01	5.34 ± 0.74	W = 0.006
1 Week	5.48 ± 0.86	5.48 ± 0.62	5.37 ± 0.64	5.21 ± 0.96	5.39 ± 0.74	T × W =0.58
4 Weeks	5.27 ± 0.86	5.34 ± 0.57	5.19 ± 0.71	5.14 ± 1.05	5.24 ± 0.76	T × W × G = 0.70
**Total work** (J)						
**Wingate 1**						
Baseline	11,467 ± 1,048	10,476 ± 1,499	10,764 ± 1,420	9,541 ± 2,262	10,591 ± 1,653	T = 0.97
1 Week	11,793 ± 1,438	10,719 ± 1,531	11,081 ± 1,726	9,566 ± 1,422	10,826 ± 1,660	G = 0.07
4 Weeks	11,494 ± 1,430	10,561 ± 1,223	10,437 ± 2,071	10,152 ± 1,698	10,674 ± 1,621	T × G = 0.33
**Wingate 2**	G × W = 0.92					
Baseline	10,565 ± 1,862	9,878 ± 1,678	9,545 ± 1,235	8,939 ± 1,800	9,761 ± 1,678	W = 0.02
1 Week	10,363 ± 1,767	9,986 ± 1,617	9,903 ± 1,605	8,220 ± 1,673	9,892 ± 1,633	T × W =0.48
4 Weeks	10,019 ± 1,785	9,835 ± 1,320	9,548 ± 1,556	9,168 ± 2,041	9,663 ± 1,619	T × W × G = 0.66
**Rate of fatigue** (%)						
**Wingate 1**						
Baseline	107.4 ± 13.9^d^	104.1 ± 14.3^d^	103.7 ± 21.0	92.4 ± 9.4^ab^	102.3 ± 15.6	T = 0.40
1 Week	105.8 ± 14.1	102.0 ± 9.9	96.2 ± 16.6	108.4 ± 9.4†	102.9 ± 13.1	G = 0.09
4 Weeks	109.8 ± 10.9^d^	103.4 ± 10.3	104.2 ± 15.9	93.1 ± 12.4^a^	102.7 ± 13.1	T × G = 0.52
**Wingate 2**						G × W = 0.96
Baseline	91.7 ± 12.0†	101.9 ± 11.4	96.0 ± 14.8	92.9 ± 13.7	95.9 ± 13.0	W = 0.02
1 Week	102.4 ± 9.5	97.9 ± 13.5	99.6 ± 11.2	90.3 ± 21.0	97.8 ± 14.1	T × W = 0.66
4 Weeks	108.5 ± 13.8‡^bd^	95.3 ± 16.6^ad^	99.6 ± 15.9^d^	82.8 ± 11.1^abc^	99.1 ± 15.2	T × W × G_q_ = 0.04

Data are means ± SD. BA signifies beta-alanine only group; BAC represents beta-alanine and creatine combined group; CRE represents the creatine only group; PLA represents the placebo group; T represents time p-level; G represents group p-level, W represents Wingate p-level, T × G represents time by group interaction, T × W × G represents time × group × Wingate interactions, _q_ represents quadratic p-level. MANOVA analysis revealed overall Wilks’ Lambda time (p = 0.004), T × G (0.65), T × W (0.97), and T × G × W effects (p = 0.21). Univariate ANOVA p-levels from MANOVA analysis are presented for each variable. † represents p < 0.05 difference from Wingate 1 baseline while ‡ represents p < 0.05 difference from Wingate 2 baseline. ^a^represents p < 0.05 difference from BA group. ^b^represents p < 0.05 difference from BAC group. ^c^represents p < 0.05 difference from CRE group. ^d^represents p < 0.05 difference from PLA group. B

### Effect size analysis

Results from effect size calculations (Cohen’s *d*) are presented in Table 6. Cohen’s *d* effect size calculations were performed to compare supplementation group means to placebo after four weeks of supplementation to assess magnitude of effects. In comparison to placebo responses, BA supplementation resulted in large effects sizes for muscle carnosine (−2.00), muscle creatine (1.16), and rate of fatigue after the first (−1.44) and second (−1.25) Wingate test; BAC supplementation resulted in large effect sizes for muscle carnosine (−1.73) and rate of fatigue following the first Wingate test (−0.90); while CRE supplementation resulted in large effect sizes for changes in muscle creatine (−0.83) and moderate effect sizes for changes in muscle carnosine (−0.75), fat free mass (−0.69), and peak power during the first Wingate test (−0.83). These findings indicate that follow-up study with a larger sample size may reveal additional statistically significant findings among groups in these variables.

**Table 6:** **Effect size calculations**

	**BA**	**BAC**	**CRE**
**Carnosine**	−2.00 (large)	−1.73 (large)	−0.75 (moderate)
**Creatine**	1.16 (large)	−0.30 (low)	−0.83 (large)
**Phosphocreatine**	−0.39 (low)	0.55 (low)	0.12 (low)
**Total Creatine**	0.77 (moderate)	−0.22 (low)	0.08 (low)
**Body Weight**	−0.63 (moderate)	−0.29 (low)	−0.33 (low)
**Fat Mass**	−0.37 (low)	0.02 (low)	0.15 (low)
**Fat Free Mass**	−0.36 (low)	−0.46 (low)	−0.69 (moderate)
**Percent Body Fat**	−0.43 (low)	0.12 (low)	0.34 (low)
**VO**_**2peak**_	−0.47 (low)	−0.02 (low)	0.38 (low)
**Max Time**	−0.76 (moderate)	−0.16 (low)	0.14 (low
**Ventilatory Threshold**	−0.34 (low	−0.4 (low)	−0.11 (low)
**Peak Lactate**	−0.58 (moderate)	0.38 (low)	−0.18 (low)
**Lactate Threshold**	−0.05 (low)	−0.12 (low)	−0.28 (low
**Peak Power Wingate 1**	−0.36 (low)	0.21 (low)	0.11 (low)
**Peak Power Wingate 2**	−0.80 (moderate)	−0.39 (low)	−0.83 (moderate)
**Total Work Wingate 1**	−0.85 (large)	−0.28 (low)	0.15 (low)
**Total Work Wingate 2**	−0.44 (low)	−0.39 (low)	−0.21 (low)
**Rate of Fatigue Wingate 1**	−1.44 (large)	−0.90 (large)	−0.78 (low)
**Rate of Fatigue Wingate 2**	−1.25 (large	−0.17 (low)	−0.49 (low

Cohen’s d calculations compared each group mean to PLA.

All calculations used data from week 4.

## Discussion

The present study sought to determine whether co-supplementation of creatine monohydrate and β-ALA would provide additive ergogenic benefits on body composition, aerobic and/or anaerobic exercise performance in recreationally-active females. We hypothesized that co-supplementation of β-ALA and creatine monohydrate may lead to greater ergogenic and performance adaptations by synergistically enhancing anaerobic threshold, aerobic capacity, time to exhaustion, and/or the ability to perform repeated 30-second sprints. Results revealed that although some benefits were found from β-ALA and creatine supplementation, there appeared to be little additive benefits from co-supplementation in recreationally active women. The following provides additional assessment of results observed.

### Muscle carnosine and phosphagens

Harris and colleagues [3] reported that β-ALA supplementation (3.2 g/day) resulted in a 42% increase in muscle carnosine levels after four weeks of supplementation. Results in the present study showed a mean increase in muscle carnosine levels of 35.3 ± 45% following BA supplementation and 42.5 ± 99% following BAC supplementation with average doses of 6.1 ± 0.7 g/day of β-ALA. While these mean changes in muscle carnosine levels following β-ALA supplementation are consistent with values reported in other studies [3,10,37-40] and we found some group effects with large effect sizes, no statistically significant interactions were observed among groups in muscle carnosine levels. The lack of statistical significance was apparently due to the large variability in muscle carnosine levels observed in response to β-ALA supplementation, assay variability, and/or inadequate sample size. More research is needed to determine the effects of β-ALA supplementation on muscle carnosine levels in recreationally-active women.

In terms of muscle phosphagen changes, it is important to note that the sample size for muscle creatine and phosphagen assessment was quite small due to prioritizing muscle carnosine assays as well as some samples not being large enough to run the appropriate assays. Therefore, statistical power is relatively low on these data. The creatine dosages used in the present study (0.3 g/kg/day of creatine for week 1 and 0.1 g/kg/day for weeks 2–4) are similar to those used with previous studies that indicate significant increases in muscle creatine after a loading and maintenance phase [19,21]. Results from the present study found non-significant increases in muscle creatine (+21%, +9.4%) and total creatine content (+6.5%, +14.5%) following creatine and β-ALA plus creatine supplementation after 1-week of loading and 3-weeks of maintenance doses, respectively. While overall results were not statistically significant, mean changes observed support previous studies that have reported that creatine loading (e.g., 20 g/day or 0.3 g/kg/d for 5 to 7-days) results in an increase in muscle creatine content by 10–40% [16,19-21,31,41] and a large effect size was observed following creatine supplementation. While the lack of significance may have simply been a result of the small sample size, it is also known that there is individual variability in response to creatine supplementation [19-21]. Additionally, measurement of muscle PCr levels can be challenging.

It is also possible that sex may have played a role in response to creatine and/or β-ALA supplementation. In this regard, most studies on creatine and β-ALA supplementation have been conducted on males and there is some evidence that females may respond differently to creatine and/or β-ALA supplementation. For example, Fosberg and colleagues [42] reported that females had greater total creatine amounts relative to tissue weight; however, other studies show there is no difference between males and females [40,43]. There are also some data suggesting that men may have greater muscle carnosine levels than women [4,44]; however, a recent study showed sex did not have an effect on increasing carnosine levels with supplementation [40]. Additionally, Bex and coworkers [45] reported that carnosine loading is more pronounced in trained versus untrained individuals. Thus, it is possible that sex and/or the types and/or amounts of training performed among participants may have influenced response to creatine and/or β-ALA supplementation.

### Body composition

As expected, body weight and markers of body composition improved over time during training in all groups. However, no significant differences were observed among groups. These findings support findings that females may experience less changes in body mass and/or fat free mass in response to creatine supplementation during training than is typically observed in men [16]. Present findings also support Kendrick et al. [39] who found that 10-weeks of β-ALA supplementation (6.4 g/day) had no effects on body composition. However, Hoffman and associates [22] reported that supplementation of β-ALA with creatine promoted greater gains in lean body mass compared to creatine alone in male strength/power athletes. Smith and colleagues [12] reported that beta alanine supplementation during high-intensity interval training in men promoted improvements in exercise capacity and lean body mass. Additionally, Kern and Robinson [46] reported that β-ALA supplementation (4 g/day for 8 weeks) in college wrestlers and football players augmented performance and increased lean mass accrual. The reason for the inconsistency in results observed on body composition in the present study remain unclear but may be due to differences in sex and/or training programs among participants.

### Aerobic exercise performance

Creatine supplementation has been purported to provide a mild effect on aerobic exercise capacity possibly through an increase in anaerobic threshold although the literature is mixed on this relationship [16]. Baguet et al. [9], used a similar supplementation protocol for β-ALA as the present study with physically active males and found no effects on VO_2peak_ as a result of supplementation. Stout and colleagues [13] measured the effects of β-ALA supplementation on VT in females. They supplemented for 28 days and found that VT and time to exhaustion were increased in the β-ALA group. The present study was unable to show similar results with β-ALA supplementation groups. There was a slight trend with the creatine only group towards improvement in time to VO_2peak_, but this was not statistically significant. The lack of significance in the present study could be due to differences in training programs among participants and/or low power and effect size of the data. It is unlikely that familiarity was a major factor as all participants underwent familiarization tests on the cycle ergometer prior to starting the study protocol.

### Blood lactate and lactate threshold

The increase in muscle carnosine following supplementation would theoretically reduce blood lactate levels during submaximal exercise and/or increase LT since one of the main functions of carnosine is as an intramuscular pH buffer. Although the carnosine results between groups in the present study were not significant, the groups supplemented with β-ALA showed greater percent changes compared to those without. The percent increase also compares closely to previous studies with significant results [3], therefore some inferences can be made based on this trend. The present study found a significant difference in peak lactate achieved during the maximal aerobic capacity test for the group supplementing with β-ALA over the combined supplementation and placebo. However, the study failed to show any differences with LT between the groups, only a trend of β-ALA supplementation improving levels after one week. Previous studies have reported mixed results pertaining to the effect of β-ALA and creatine supplementation on blood lactate accumulation and LT. Van Thienen and colleagues [47] reported no difference between groups in blood lactate levels in healthy males after an incremental maximal cycle ergometer test followed by a 30 second all out sprint after eight weeks of supplementation with β-ALA (2–3 g/d weeks 1–4 and 4 g/d for weeks 4–8) or placebo. Zoeller et al. [15] studied 55 men who supplemented with β-ALA (3–6 g/d), creatine (5 g/d), a combination or placebo for 28 days and reported a greater VO_2peak_ at LT for the combined supplementation group, suggesting that this supplementation protocol may delay the onset of LT during incremental exercise.

The present study may have failed to show improvements in lactate accumulation and LT with β-ALA alone or the combined β-ALA and creatine supplementation strategy for various reasons. First, the power analysis and effect size calculations were low, which indicates the strength of the data could be improved, possibly with a larger sample size. Also, the present study examined the effects of supplementation in recreationally active females, who did not engage in a standardized training program during the four weeks of the study. Perhaps with a training program, like one seen in other studies, there may have been training effects seen for lactate variables. Finally, since we did not observe statistically significant differences in muscle carnosine or phosphagen levels due to large variation in response to the supplementation protocol, variability in measurement, and/or low statistical power; it is possible that this may have limited the potential ergogenic benefit.

### Anaerobic exercise performance

Creatine supplementation has been consistently reported to increase anaerobic sprint and/or exercise capacity [16]. For example, Kreider and colleagues [48] reported that creatine supplementation (15 g/day for 28-days) significantly increased repetitive sprint performance and muscle mass during training in college football players. Wiroth and colleagues [49] reported that creatine supplementation improved maximal power and work during a set of 5 × 10 second sprints on the cycle ergometer. Green and colleagues [50] reported that creatine supplementation (20 g/day for 6-days) increased PP during the first arm Wingate test and the decline in performance was less after the second leg Wingate test. Ziegenfuss et al. [51] also showed that creatine supplementation in college athletes resulted in increased TW and PP during multiple maximal 10-sec sprints on a cycle ergometer. Results from the present study, however, did not reveal an ergogenic benefit when performing repeated 30-sec anaerobic capacity tests. These findings may be related to the length of the sprint which is generally more dependent on glycolytic capacity rather than phosphagen availability.

A number of studies have also reported that β-ALA supplementation provides ergogenic benefit during high intensity exercise [1,2,5,44,47]. For example, Hill and colleagues [10] reported that 10-weeks of β-ALA supplementation significantly increased total work performed during high-intensity cycling by approximately 13%. Van Thienen et al. [47] reported that β-ALA supplementation (2.4 g/day for 8-weeks) significantly increased sprint performance at the end of an exhausted endurance cycling exercise bout. Hoffman et al. [52] reported that β-ALA supplementation (4.5 g/day for 3-weeks) in college football players tended to decrease fatigue rate during sprint exercise (p = 0.07). Another study by this group [22] examined the effects of creatine alone, β-ALA and creatine combined and placebo supplementation for 10-weeks in strength power athletes. The researchers reported some beneficial effects on improvements in strength and lean tissue accretion from β-ALA and/or creatine supplementation. Tobias et al. [53] reported that β-ALA supplementation (6.4 g/day for 4-weeks) significantly improved repetitive anaerobic capacity while performing 4 × 30-sec sprints in judo and jujitsu athletes. Similarly, De Salles Painelli and coworkers [54] reported that β-ALA supplementation (6.4 g/day for 4-weeks) significantly improved anaerobic capacity while performing 4 × 30-sec sprints in untrained and trained men. In the present study, there was some evidence that β-ALA supplementation may have led to an improvement in rate of fatigue after four weeks of supplementation. However, we did not find that β-ALA with creatine supplementation improved repetitive bouts of 30-sec sprint performance in recreationally active women. It is possible that greater benefits may have been observed from performing more than two 30-sec Wingate sprints as noted above and/or longer sprints. In this regard, Hobson et al. [11] reported that β-ALA supplementation was effective in improving sprint performance in tasks lasting longer than 60-seconds. Saunders et al. [55] tested this theory in a study utilizing the YoYo Intermittent Recovery Test in participants supplementing with β-ALA for 12 weeks. This test is designed to assess the ability to perform and recover from multiple sprints, as seen in many sports. They found supplementation improved performance on this test and suggested it was due to enhanced muscle buffering capabilities between bouts of high intensity exercise resulting from the increased muscle carnosine due to supplementation with β-ALA [55]. However, more research is needed to examine the potential ergogenic value of β-ALA with and without creatine monohydrate supplementation in this population.

## Conclusion

This is one of the first studies to use a more individualized dosing strategy for β-ALA supplementation instead of providing a standardized amount β-ALA for all participants irrespective of difference in body mass. Although the mean increases in muscle carnosine levels were similar to those reported in the literature, changes in muscle carnosine levels were not statistically increased in the present study. The lack of significance may have been due to the dosing strategy employed in that the calculated doses may not have been great enough to elicit positive responses. Additionally, there may be a sex effect with females needing a different amount of β-ALA to consistently increase muscle carnosine levels compared to males. Further, the small sample size of the present study resulting in low power and effect sizes in some instances may have contributed to the lack of significant findings as previous research has demonstrated that four weeks of creatine and β-ALA supplementation was sufficient to increase muscle carnosine and phosphagen levels. Moreover, results of the present study did not show supplementation to have significant effects on body composition, aerobic or anaerobic performance measures. However, perhaps a greater total amount of β-ALA is needed to be ingested over time in women in order for performance adaptations to occur, especially without the addition of a standardized exercise training program. Further studies should be conducted to examine the potential independent and synergistic effects of a combined supplementation of creatine, β-ALA, and other purported nutritional ergogenic aids in untrained and trained male and female populations. Additionally, future studies should examine the effects of combined supplementation on muscle carnosine and phosphagen levels in a larger and/or more active population.

## References

[1] Culbertson JY, Kreider RB, Greenwood M, Cooke M (2010). Effects of beta-alanine on muscle carnosine and exercise performance: a review of the current literature. Nutrients.

[2] Harris RC, Sale C (2012). Beta-alanine supplementation in high-intensity exercise. Med Sport Sci.

[3] Harris RC, Tallon MJ, Dunnett M, Boobis L, Coakley J, Kim HJ, Fallowfield JL, Hill CA, Sale C, Wise JA (2006). The absorption of orally supplied beta-alanine and its effect on muscle carnosine synthesis in human vastus lateralis. Amino Acids.

[4] Harris RC, Wise JA, Price KA, Kim HJ, Kim CK, Sale C (2012). Determinants of muscle carnosine content. Amino Acids.

[5] Quesnele JJ, Laframboise MA, Wong JJ, Kim P, Wells GD (2014). The effects of beta-alanine supplementation on performance: a systematic review of the literature. Int J Sport Nutr Exerc Metab.

[6] Sale C, Artioli GG, Gualano B, Saunders B, Hobson RM, Harris RC (2013). Carnosine: from exercise performance to health. Amino Acids.

[7] Sale C, Saunders B, Harris RC (2010). Effect of beta-alanine supplementation on muscle carnosine concentrations and exercise performance. Amino Acids.

[8] Stellingwerff T, Decombaz J, Harris RC, Boesch C (2012). Optimizing human in vivo dosing and delivery of beta-alanine supplements for muscle carnosine synthesis. Amino Acids.

[9] Baguet A, Koppo K, Pottier A, Derave W (2010). Beta-alanine supplementation reduces acidosis but not oxygen uptake response during high-intensity cycling exercise. Eur J Appl Physiol.

[10] Hill CA, Harris RC, Kim HJ, Harris BD, Sale C, Boobis LH, Kim CK, Wise JA (2007). Influence of beta-alanine supplementation on skeletal muscle carnosine concentrations and high intensity cycling capacity. Amino Acids.

[11] Hobson RM, Saunders B, Ball G, Harris RC, Sale C (2012). Effects of beta-alanine supplementation on exercise performance: a meta-analysis. Amino Acids.

[12] Smith AE, Walter AA, Graef JL, Kendall KL, Moon JR, Lockwood CM, Fukuda DH, Beck TW, Cramer JT, Stout JR (2009). Effects of beta-alanine supplementation and high-intensity interval training on endurance performance and body composition in men; a double-blind trial. J Int Soc Sports Nutr.

[13] Stout JR, Cramer JT, Zoeller RF, Torok D, Costa P, Hoffman JR, Harris RC, O'Kroy J (2007). Effects of beta-alanine supplementation on the onset of neuromuscular fatigue and ventilatory threshold in women. Amino Acids.

[14] Jordan T, Lukaszuk J, Misic M, Umoren J (2010). Effect of beta-alanine supplementation on the onset of blood lactate accumulation (OBLA) during treadmill running: Pre/post 2 treatment experimental design. J Int Soc Sports Nutr.

[15] Zoeller RF, Stout JR, O'Kroy JA, Torok DJ, Mielke M (2007). Effects of 28 days of beta-alanine and creatine monohydrate supplementation on aerobic power, ventilatory and lactate thresholds, and time to exhaustion. Amino Acids.

[16] Kreider RB (2003). Effects of creatine supplementation on performance and training adaptations. Mol Cell Biochem.

[17] Gaitanos GC, Williams C, Boobis LH, Brooks S (1993). Human muscle metabolism during intermittent maximal exercise. J Appl Physiol (1985).

[18] Sahlin K (1992). Metabolic factors in fatigue. Sports Med.

[19] Greenhaff PL, Bodin K, Soderlund K, Hultman E (1994). Effect of oral creatine supplementation on skeletal muscle phosphocreatine resynthesis. Am J Physiol.

[20] Harris RC, Soderlund K, Hultman E (1992). Elevation of creatine in resting and exercised muscle of normal subjects by creatine supplementation. Clin Sci (Lond).

[21] Hultman E, Soderlund K, Timmons JA, Cederblad G, Greenhaff PL (1996). Muscle creatine loading in men. J Appl Physiol (1985).

[22] Hoffman J, Ratamess N, Kang J, Mangine G, Faigenbaum A, Stout J (2006). Effect of creatine and beta-alanine supplementation on performance and endocrine responses in strength/power athletes. Int J Sport Nutr Exerc Metab.

[23] Kendrick IP, Kim HJ, Harris RC, Kim CK, Dang VH, Lam TQ, Bui TT, Wise JA (2009). The effect of 4 weeks beta-alanine supplementation and isokinetic training on carnosine concentrations in type I and II human skeletal muscle fibres. Eur J Appl Physiol.

[24] Smith AE, Moon JR, Kendall KL, Graef JL, Lockwood CM, Walter AA, Beck TW, Cramer JT, Stout JR (2009). The effects of beta-alanine supplementation and high-intensity interval training on neuromuscular fatigue and muscle function. Eur J Appl Physiol.

[25] Smith AE, Stout JR, Kendall KL, Fukuda DH, Cramer JT (2012). Exercise-induced oxidative stress: the effects of beta-alanine supplementation in women. Amino Acids.

[26] Walter AA, Smith AE, Kendall KL, Stout JR, Cramer JT (2010). Six weeks of high-intensity interval training with and without beta-alanine supplementation for improving cardiovascular fitness in women. J Strength Cond Res.

[27] Bergstrom J (1975). Percutaneous needle biopsy of skeletal muscle in physiological and clinical research. Scand J Clin Lab Invest.

[28] Almada AL, Kreider RB, Ransom J, Rasmussen C, Tutko R, Milnor P (1999). Comparison of the reliability of repeated whole body DEXA scans to repeated spine and hip scans. J Bone Miner Res.

[29] Crouter SE, Antczak A, Hudak JR, DellaValle DM, Haas JD (2006). Accuracy and reliability of the ParvoMedics TrueOne 2400 and MedGraphics VO2000 metabolic systems. Eur J Appl Physiol.

[30] Tanner RK, Fuller KL, Ross ML (2010). Evaluation of three portable blood lactate analysers: Lactate Pro, Lactate Scout and Lactate Plus. Eur J Appl Physiol.

[31] Jagim AR, Oliver JM, Sanchez A, Galvan E, Fluckey J, Riechman S, Greenwood M, Kelly K, Meininger C, Rasmussen C, Kreider RB (2012). A buffered form of creatine does not promote greater changes in muscle creatine content, body composition, or training adaptations than creatine monohydrate. J Int Soc Sports Nutr.

[32] Harris RC, Hultman E, Nordesjo LO (1974). Glycogen, glycolytic intermediates and high-energy phosphates determined in biopsy samples of musculus quadriceps femoris of man at rest. Methods and variance of values. Scand J Clin Lab Invest.

[33] Soderlund K, Hultman E (1986). Effects of delayed freezing on content of phosphagens in human skeletal muscle biopsy samples. J Appl Physiol (1985).

[34] Tarnopolsky MA, Parise G (1999). Direct measurement of high-energy phosphate compounds in patients with neuromuscular disease. Muscle Nerve.

[35] Dunnett M, Harris RC (1997). High-performance liquid chromatographic determination of imidazole dipeptides, histidine, 1-methylhistidine and 3-methylhistidine in equine and camel muscle and individual muscle fibres. J Chromatogr B Biomed Sci Appl.

[36] Twisk J, de Vente W (2002). Attrition in longitudinal studies. How to deal with missing data. J Clin Epidemiol.

[37] Baguet A, Bourgois J, Vanhee L, Achten E, Derave W (2010). Important role of muscle carnosine in rowing performance. J Appl Physiol (1985).

[38] Derave W, Ozdemir MS, Harris RC, Pottier A, Reyngoudt H, Koppo K, Wise JA, Achten E (2007). beta-Alanine supplementation augments muscle carnosine content and attenuates fatigue during repeated isokinetic contraction bouts in trained sprinters. J Appl Physiol (1985).

[39] Kendrick IP, Harris RC, Kim HJ, Kim CK, Dang VH, Lam TQ, Bui TT, Smith M, Wise JA (2008). The effects of 10 weeks of resistance training combined with beta-alanine supplementation on whole body strength, force production, muscular endurance and body composition. Amino Acids.

[40] Stegen S, Bex T, Vervaet C, Vanhee L, Achten E (2014). Derave W: **beta-Alanine dose for maintaining moderately elevated muscle carnosine levels**. Med Sci Sports Exerc.

[41] Buford TW, Kreider RB, Stout JR, Greenwood M, Campbell B, Spano M, Ziegenfuss T, Lopez H, Landis J, Antonio J (2007). International Society of Sports Nutrition position stand: creatine supplementation and exercise. J Int Soc Sports Nutr.

[42] Forsberg AM, Nilsson E, Werneman J, Bergstrom J, Hultman E (1991). Muscle composition in relation to age and sex. Clin Sci (Lond).

[43] Balsom PD, Soderlund K, Sjodin B, Ekblom B (1995). Skeletal muscle metabolism during short duration high-intensity exercise: influence of creatine supplementation. Acta Physiol Scand.

[44] Derave W, Everaert I, Beeckman S, Baguet A (2010). Muscle carnosine metabolism and beta-alanine supplementation in relation to exercise and training. Sports Med.

[45] Bex T, Chung W, Baguet A, Stegen S, Stautemas J, Achten E, Derave W (2014). Muscle carnosine loading by beta-alanine supplementation is more pronounced in trained vs. untrained muscles. J Appl Physiol (1985).

[46] Kern BD, Robinson TL (2011). Effects of beta-alanine supplementation on performance and body composition in collegiate wrestlers and football players. J Strength Cond Res.

[47] Van Thienen R, Van Proeyen K, Vanden Eynde B, Puype J, Lefere T, Hespel P (2009). Beta-alanine improves sprint performance in endurance cycling. Med Sci Sports Exerc.

[48] Kreider RB (1998). Creatine supplementation: analysis of ergogenic value, medical safety, and concerns. J Exerc Physiol Online.

[49] Wiroth JB, Bermon S, Andrei S, Dalloz E, Hebuterne X, Dolisi C (2001). Effects of oral creatine supplementation on maximal pedalling performance in older adults. Eur J Appl Physiol.

[50] Green JM, McLester JR, Smith JE, Mansfield ER (2001). The effects of creatine supplementation on repeated upper- and lower-body Wingate performance. J Strength Cond Res.

[51] Ziegenfuss TN, Rogers M, Lowery L, Mullins N, Mendel R, Antonio J, Lemon P (2002). Effect of creatine loading on anaerobic performance and skeletal muscle volume in NCAA Division I athletes. Nutrition.

[52] Hoffman JR, Ratamess NA, Faigenbaum AD, Ross R, Kang J, Stout JR, Wise JA (2008). Short-duration beta-alanine supplementation increases training volume and reduces subjective feelings of fatigue in college football players. Nutr Res.

[53] Tobias G, Benatti FB, de Salles PV, Roschel H, Gualano B, Sale C, Harris RC, Lancha AH, Artioli GG (2013). Additive effects of beta-alanine and sodium bicarbonate on upper-body intermittent performance. Amino Acids.

[54] de Salles PV, Saunders B, Sale C, Harris RC, Solis MY, Roschel H, Gualano B, Artioli GG, Lancha AH (2014). Influence of training status on high-intensity intermittent performance in response to beta-alanine supplementation. Amino Acids.

[55] Saunders B, Sunderland C, Harris RC, Sale C (2012). beta-alanine supplementation improves YoYo intermittent recovery test performance. J Int Soc Sports Nutr.

